# Supplementary material for: Why does the number of antenatal care visits in Ethiopia remain low?: A Bayesian multilevel approach
Source: PLoS One. 2024 May 3;19(5):e0302560. doi: 10.1371/journal.pone.0302560 (PMC11068190; doi:10.1371/journal.pone.0302560)
Supplement: Attachment — Submitted filename: plos_R1.docx [file pone.0302560.s003.docx]

**Why does the number of antenatal care visits in Ethiopia remain low? A Bayesian multilevel approach**

Daniel Atlaw^1*^, Tesfaye Getachew Charkos^2^, Jeylan Kasim^1^, Vijay Kumar Chatu^3,4^

^1^ Public Health Department, School of Health Science, Madda Walabu University, Bale Goba, Ethiopia

^2^ School of Public Health, Adama Hospital Medical College, Adama, Ethiopia

^3^Center for Transdisciplinary Research, Saveetha Dental College, Saveetha Institute of Medical and Technical Sciences (SIMATS), Saveetha University, Chennai-600077, India; email: vijay.chattu@mail.utoronto.ca

^4^Center for Evidence-based Research, Global Health Research and Innovations Canada (GHRIC), Toronto, ON, Canada.

***Address for Correspondence***

Daniel Atlaw

Phone: +251911545869

Email: danielatmwu@gmail.com

Dr. Tesfaye Getachew Charkos

Phone: +251 912270052

Email: [tesfayegch@gmail.com](mailto:tesfayegch@gmail.com)

**Abstract**

**Introduction:** Antenatal care (ANC) visit is a proxy for maternal and neonatal health. The ANC is a key indicator of access and utilization of health care for pregnant women. Recently, eight times ANC visits have been recommended during the pregnancy period. However, there are still less frequent ANC visits experienced by Ethiopian women. Therefore, the objective of this is identify associated factors of the number of ANC visits in Ethiopia.

**Methods:** A community-based cross-sectional study design was conducted from March 21 to June 28/2019. Data were collected using interviewer-admistered questionare from reproductive age group. A stratified cluster sampling was used to select enumeration areas, households, and women from selected households. A Bayesian multilevel negative binomial model was applied for analysis of this study. There is intra-class correlation (ICC)=23.42% and 25.51% for the null and final model, respectively. Data were analyzed using the STATA version 17.0. The adjusted incidence risk ratio (IRR) with 95% credible intervals (CrI) was used to decleare association.

**Result**: A total of 3915 pregnant women were included in this study. The average age of the participants was 28.7 years. Nearly one-fourth (26.5 %) of pregnant women did not have ANC visits, and 3% had eight-time ANC visits in Ethiopia. In the adjusted model, the age of the women 25-28 years (IRR:1.13; 95% CrI: 1.11, 1.16), 29-33 years (IRR: 1.15; 95% CrI: 1.15, 1.16), ≥34 years (IRR:1.14; 95% CrI: 1.12, 1.17), being a primary school (IRR: 1.22, 95% CrI: 1.21, 1.22), secondary school and above (IRR: 1.26, 95% CrI: 1.26, 1.26), women delivered in health facility (IRR: 1.93; 95% CrI: 1.92, 1.93), delivered with cesarian section (IRR: 1.18; 95% CrI: 1.18, 1.19), multiple (twin) pregnancy (IRR: 1.11; 95% CrI: 1.10, 1.12), richest (IRR:1.23; 95% CrI: 1.23, 1.24), rich family (IRR: 1.34, 95% CrI: 1.30, 1.37), middle income (IRR: 1.29, 95% CrI: 1.28, 1.31), poor family (IRR = 1.28, 95% CrI:1.28, 1.29), households with total family size of ≥ 5 (IRR: 0.92; 95% CrI: 0.91, 0.92), and being a rural resident (IRR: 0.92, 95% CrI: 0.92, 0.94) were shown to have a significant association with the number of ANC visits.

**Conclusion:** Overall, 26.5 % of pregnant women do not have ANC visits during their pregnancy, and 3% of women have eight-time ANC visits. The main factors determined in this study were maternal age, educational level, wealth index, and place of residence were significantly associated with the number of ANC visits in Ethiopia.

**Keywords**: Antenatal care visits, Pregnant, Bayesian, Poisson, Ethiopia

**Introduction**

Antenatal care (ANC) visit is a proxy for maternal and neonatal health. The ANC visit is a key indicator of access to and utilization of health care for pregnant women. It is one of important compenent of maternal and child health care service for reducing maternal and child mortality rates (1,2). Early initiation and increasing the encouraging regular ANC visits are important to reduce pregnancy complications (3). For instance, a systematic review in Ethiopia revealed that a focused quality ANC significantly reduces neonatal mortality by 34% (4). The main objective of ANC is the early identification of preexisting diseases or risk factors that occur during pregnancy and childbirth, as well as promoting well-being of mather and their newborn (4–6). The ANC package is important to prepare for birth and avoid threats affecting mothers and babies during pregnancy (7).

About 15% of pregnancies result in life-threatening complications that requires intervention by skilled health care providers (8). In middle and low-income countries, complications during pregnancy and childbirth are major causes of death and disability among reproductive age women (9,10). To decrease the avoidable causes of maternal and infant mortality, WHO recommended a minimum of eight or more ANC follow-up visits (11). However, in Africa, women lack access to health facilities (why? At least state one main reason as the sentence reads incomplete) (12). The utilization of eight or more ANC visits is only 6.8% in Sub-Saharan Africa (13). The benefit of ANC is determined number of ANC visits and the components of services provided during visits (14).

About 32% of pregnant women did not receive ANC visits throughout their pregnancy period in Ethiopia (11). Previous studies revealed that as the number of ANC visits increases, maternal and neonatal health were shown to improve in Ethiopia (15). In Ethiopia, although ANC servise coverage was increased, yet the recommended number of ANC visit is not achieved at national level (15). To increase the number of ANC visits, it is important to identify factors that affect ANC visits in Ethiopia.

Different studies were conducted in Ethiopia, but the previous studies were conducted on utilization level (16,17), time of initiation (3,18–21), and associated factors (3,12,20) rather than focusing on the number of visits, which may result in loss of information in classification count data. Therefore, this study, aimed to identify community and individual-level determinants of the number of ANC visits in Ethiopia.

**Methods**

**Study Setting and Design**

Ethiopia is one of the East African countries,which has 10 regional states (Afar, Amhara, Benishangul-Gumuz, Gambella, Oromia, Somali, Southern Nations, Nationalities and People’s (SNNP), Harari, Sidama, and Tigray), and two metropolis towns (Addis Ababa and Dire Dawa). It is estimated that the total population of Ethiopia is 105,163,988 (22). Of this, 52,439,998 are women of reproductive age (22). Ethiopian Ministry of Health provides maternal health service free of charge. A cross-sectional community-based study design was conducted from March 21 to June 28 /2019, among reproductive age women in Ethiopia.

**Data source and sampling**

The Ethiopian Demographic and Health Survey (EDHS) data are collected nationally every 5 years on key indicators. Data were collected using an interviewer-administered questionnaire from reproductive age (15–49 years) women. The questionnaire includes socio-demographic, socioeconomic, and service related to maternal health variables (23). The source of data for this analysis was from DHS website ([www.dhsprogram.com](http://www.dhsprogram.com)) after requesting permission by submitting protocol for the study.

A two-stage stratified cluster sampling was employed to select total of 305 enumeration areas (EAs) using propability proportional to EA size. Out of which 93 were urban and 212 were rural areas. Independent selection was applied in each sampling stratum (23).

Then a fixed number of 30 housholds per cluster were selected using systematic sampling. From each selected housholds all reproductive age women were eligible for interview. About 8885 women were interviewed of which 3962 women were delivered within 5 years before survey and they are interviewed for ANC visits (23). From 3962 women interviewed 3915 were included for analysis of this study while 47 women were excluded due to missed information on outcome of interest (Figure 1).

**Study variables and measurements**

The response variable of this study was the number of ANC visits. Both community and individual variables are considered as predictor variables. Community water source classified as an improved and unimproved water source, place of residence (urban and rural), and region (the ten Ethiopian regions were recoded into three larger categories agrarian, pastoralist, and metropolis). Four regions namely (Tigray, Amhara, Oromia, and Southern Nations, Nationalities People's Region (SNNPR)) were recoded into the agrarian region. Three regions namely (Afar, Somali, Benishangul, and Gambella) were recoded as pastoralist region. While, Harari, Addis Ababa, and Dire Dawa are among the metropolises' administration regions.

The individual**-**level variables: Age (maternal age was categorized by using quartile in years interval as follows: ≤24 first quartile, 25–28 second quartile, 29–33 third quartile, and ≥ 34 fourth quartile), level of education (no education, primary education, and secondary and above), birth order (1st, 2nd–4th, and ≥5th), wealth index. Household income is classified using the wealth index with five catogories (poorest, poorer, middle, richer, and richest), number of under-five children (0, 1-2, ≥3), family size (<5, ≥5), place of delivery (home, facility), mode of delivery (cesarian section(C/S), spontaneous vaginal delivery (SVD)), marital status (single, married, widowed and divorced and birth interval (< 24 months, 24-36 months, ≥ 36 months) and multiple pregnancies (yes, no).

**Data analysis**

Since EDHS data are hierarchical, using a standard model will affect the standard error of effect size, which in turn affects the hypothesis. Women in a cluster may have similar characteristics to those in the other cluster. This similarity within cluster will violets (?) the rule of independency of observation and equal variance across the cluster. Therefore, using multilevel model is best for this data rather than using standard model which helps to compute both fixed effect and random effect variation simultaneously.

A poison regression model is proposed to use for this study, because of dependent variable is the number of ANC visits, which is a countable variable. However, the assumption of poison regression is not met (data is over-dispersed: variance is larger than mean). Therefore, the negative binomial model is appropriate than the standard Poisson regression model.

In this four models were fitted to estimate fixed effect for indidual and community level variable and random effect for variation among cluster. Since measure of variation of cluster was significant indicating higher intraclass correlation coefficient (ICC).

A Bayesian multilevel negative binomial regression model was usedas it is not dependent on the p-value to determine whether the variable is significant or not. The p-value may lead to imprecise evidence as it depends on small size. The prior information for each coefficient of the variables assumed that normally distributed with zero mean and 10,000 variances.

The data were correlated, having intra-class correlation (ICC)=23.42% and 25.51% for the null and saturated model, respectively, since the value is greater than 5 percent correlation is significant within clusters (25). The multilevel fitted with 4 models: Model I (null model) was done without independent variables. Model II was fitted for variables at individual level. Model II was fitted for variables at individual level, and Model III was fitted for variables at community level and model IV was adjusted for individual and community level variables.. The fourth model was fitted to estimate independent effects of variables both at community and individual level on the number of ANC visits.. Adjusted incidence ratio (IRR) with 95% credible intervals (CrI) was used to declare association. Goodness of fit test was assessed using the deviance information criterion (DIC). Variance Inflation Factor (VIF) < 5 was considered to check multicollinearity between the individual and community-level variables. All analysis was using STATA version 17.0 (Stata Corp., College Station, TX, USA).

**Result**

**Characteristics of the study participants**

Of the total study subjects, 30.4% of the women were between 25-29 years old and the average age of the participants was 28.7 years. Concerning their education, more than half of the women do not have formal education (51.3%), while only about 3.8% of them attended higher education. Most of the women were married (93.9 %), from agrarian regions (87.5%), and rural areas (73.9 %) (Table 2).

Table 2	The number of antenatal care visits by sciodemographic charasterstics of women in Ethiopia from 2019 EDHS**.**

| Variable | Categories | Frequency | Percent (%) |
| --- | --- | --- | --- |
| Age of women in years | <=24 | 895 | 22.86 |
|  | 25-28 | 1,229 | 31.39 |
|  | 29-33 | 818 | 20.89 |
|  | >34 | 973 | 24.85 |
| Education level of women | No formal education | 2,049 | 52.34 |
|  | Primary | 1,281 | 32.72 |
|  | Secondary and above | 585 | 14.94 |
| Marital status of women | Married | 3,630 | 92.72 |
|  | Non-married | 285 | 7.28 |
| Region of women’s residence | Agrarian | 1,690 | 43.17 |
|  | Pastoralist | 1,423 | 36.35 |
|  | Metropolis | 802 | 20.49 |
| Residence of women | Urban | 976 | 24.93 |
|  | Rural | 2,939 | 75.07 |
| Wealth index of household | Poorest | 1,179 | 30.11 |
|  | Poor | 676 | 17.27 |
|  | Middle | 577 | 14.74 |
|  | Rich | 532 | 13.59 |
|  | Richest | 951 | 24.29 |
| Community water source | Improved | 2,646 | 67.59 |
|  | Non-Improved | 1,269 | 32.41 |
| Household family size | <5 | 1,903 | 48.61 |
|  | >=5 | 2,012 | 51.39 |
| Birth interval (preceding) | < 24 months | 718 | 23.33 |
|  | 24-36 months | 863 | 28.05 |
|  | > 36 months | 1,496 | 48.62 |
| Place of delivery | Home | 1,780 | 45.47 |
|  | Facility | 2,135 | 54.53 |
| Mode of delivery | C/S | 3,638 | 92.92 |
|  | SVD | 277 | 7.08 |
| Multiple pregnancies | No | 3,851 | 98.37 |
|  | Yes | 64 | 1.63 |

C/S- cesarian section, SVD -spontaneous vaginal

Out of the total pregnant women in this study, 26.5% do not have ANC visits during their pregnancy, 21.4 % of women have at least four ANC visits, and only 3% have at least eight ANC visits (Figure 2).

Figure 2: Number of ANC visit status of women delivered within five years from EDHS 2019

**Factors associated with the number of ANC Visits in Ethiopia**

**Individual level determinants**: - The number of ANC visits was identified to be higher among women in the age group of 25-28 years, 29-33 years, and ≥34 years than women in the group ≤24 years by 13.2% (IRR: 1.13; 95% CrI: 1.11, 1.16), (IRR: 1.15; 95% CrI: 1.15, 1.16) and (IRR:1.14: 95% CrI: 1.12, 1.17), respectively. Women who have attended primary education were shown to have an increased number of ANC visits by 22% (IRR: 1.22, 95% CrI: 1.22, 1.23) compared with those who did not have attended formal education, while attending secondary school and above shown to increase the number of ANC visit by 26% (IRR: 1.26, 95% CrI: 1.26, 1.26) (Table 3).

Women who delivered in a health facility were two times more likely to have a higher number of ANC visits compared with women who delivered at home (IRR: 1.93; 95% CrI: 1.92, 1.93). Similarly, women who delivered with cesarian section were shown to have a higher number of ANC visits compared with women delivered with spontaneous vaginal delivery by 18% (IRR: 1.18; 95% CrI: 1.18, 1.19). Women with multiple (twin) pregnancies were shown to have a higher number of ANC visits by 11% (IRR: 1.11; 95% CrI: 1.10, 1.12) compared with women who have singleton pregnancies. Concerning birth interval of preceding pregnancy women with an interval greater than 36 months were shown to have a higher number of ANC visits by 22 % (IRR: 1.22; 95% CI: 1.21, 1.23) compared with women having birth interval of less than 24 months (Table 3).

Women from households who have a total family size of ≥ 5 were shown to have decreased the number of ANC visits by 9.4% (IRR: 0.92; 95% CrI: 0.91, 0.92) compared with women from households who have a total family size < 5. Concerning the wealth index, it is revealed that women from the richest, rich, middle, and poor households were shown to have increased number of ANC visits by 28.8% (IRR:1.23; 95% CrI: 1.23, 1.24), 29.4% (IRR: 1.34; 95% CrI: 1.30, 1.37), 34.2% (IRR:1.29; 95% CrI: 1.28, 1.31) and 24% (IRR: 1.29, 95% CI:1.29, 1.30), respectively compared to women from poorest households (Table 3).

**Community level determinants: -** Women from the rural area were shown to have a reduced number of ANC visits by 8% (IRR: 0.92, 95% CI: 0.93, 0.94) compared to women from the urban area (Table 3). Similarly, women from areas with unimproved water sources were identified to have a reduced number of ANC visits by 6.8% (IRR: 0.92, 95% CI: 0.91, 0.92) compared with women from areas with improved water sources. The number of ANC visits was reduced by 22.6% (IRR: 0.77, 95% CI: 0.77, 0.78) among women from pastoralist regions compared to women from agrarian regions, while it was increased by 1% (IRR:1.01, 95% CI: 1.01, 1.04) among women from metropolis region (Table 3).

Table 3: Multivariable analysis of factors associated with the number of ANC visits from 2019 mini-demographic data in Ethiopia.

| Variable | | ModeI I (null) | Model II | Model III | Model IV |
| --- | --- | --- | --- | --- | --- |
|  | Categories |  | aIRR (CI) | aIRR (CI) | aIRR (CI) |
| Age of women in years | <=24 |  | 1 |  | 1 |
|  | 25-28 |  | 1.194 (1.185, 1.203) * |  | 1.132 (1.112, 1.156) * |
|  | 29-33 |  | 1.226 (1.219, 1.235) * |  | 1.152 (1.147, 1.156) * |
|  | >34 |  | 1.223 (1.215, 1.230) * |  | 1.144 (1.119, 1.174) * |
| Education level of women | No formal education |  | 1 |  | 1 |
|  | Primary |  | 1.231 (1.226, 1.238) * |  | 1.221 (1.218, 1.222) * |
|  | Secondary and above |  | 1.240 (1.234, 1.243) * |  | 1.261 (1.260, 1.263) * |
| Marital status of women | Married |  | 1 |  | 1 |
|  | Non-married |  | 1.057 (1.054, 1.059) * |  | 0.999 (0.996, 1.002) |
| Wealth index of household | Poorest |  | 1 |  | 1 |
|  | Poor |  | 1.341 (1.332, 1.352) * |  | 1.234 (1.227, 1.240) * |
|  | Middle |  | 1.473 (1.468, 1.480) * |  | 1.342 (1.304, 1.370) * |
|  | Rich |  | 1.455 (1.433, 1.471) * |  | 1.294 (1.285, 1.308) * |
|  | Richest |  | 1.618 (1.614, 1.622) * |  | 1.288 (1.286, 1.291) * |
| Household family size | <5 |  | 1 |  | 1 |
|  | ≥5 |  | 0.926 (0.918, 0.935) * |  | 0.916 (0.915, 0.917) * |
| Birth interval (preceding) | < 24 months |  | 1 |  | 1 |
|  | 24-36 months |  | 1.047 (1.047, 1.052) * |  | 1.056 (1.051, 1.061) * |
|  | > 36 months |  | 1.107 (1.103, 1.111) * |  | 1.223 (1.211, 1.234) * |
| Place of delivery | Home |  | 1 |  | 1 |
|  | Facility |  | 1.853 (1.848, 1.862) * |  | 1.931 (1.926, 1.933) * |
| Mode of delivery | C/S |  | 1 |  | 1 |
|  | SVD |  | 1.074 (1.064, 1.085) * |  | 1.107 (1.100, 1.116) * |
| Multiple pregnancies | No |  | 1 |  | 1 |
|  | Yes |  | 1.084 (1.073, 1.093) * |  | 1.185 (1.178, 1.195) * |
| Community water source | Improved |  |  | 1 | 1 |
|  | unimproved |  |  | 0.736 (0.694, 0.783) * | 0.932 (0.926, 0.940) * |
| Region of women’s residence | Agrarian |  |  | 1 | 1 |
|  | Pastoralist |  |  | 0.709 (0.669, 0.751) * | 0.774 (0.771, 0.779) * |
|  | metropolis |  |  | 1.021 (0.951, 1.091) * | 1.017 (1.001, 1.036) * |
| Residence of women | Urban |  |  | 1 | 1 |
|  | Rural |  |  | 0.686 (0.643, 0.733) | 0.920 (0.919, 0 .921) * |
| Interclass correlation coefficient (ICC) | | 23.42% | 37.00% | 23.01% | 24.22% |
| Deviance information criteria (DIC) | | 17009 | -614292 | -16442.88 | -614240.7 |

**aIRR- adjusted Incidence rate ratio, * p-value less than 0.05, C/S- cesarian section, SVD -spontaneous vaginal delivery**

**Discussion**

In Ethiopia, nearly one-fourth of women did not have ANC visits, and only 3% of women have eight ANC visits during the whole pregnancy, which WHO currently recommends. This finding is slightly higher than the study conducted in 2016 (26). The difference may be due to variations in the study period, and the recommendation of the new guidelines by WHO in 2016 might have contributed to the difference.

The finding from this study revealed that the number of ANC visits increases with the increasing age of women. The women in the age group of higher than 24 years were shown to have a higher count of ANC visits than women in the age group of ≤ 24 years. This finding is supported by a study conducted in Nigeria and Malawi in 2017 (27). At the same time, it is contradicted by another study conducted in Ethiopia, which revealed that the likelihood of ANC visits decreases among women in the age group of 35 to 49 years and increases among women between the age of 15 to 19 years (26). The difference might be due to the different models used for this data, and the authors used the Bayesian model, which is a more appropriate and representative sample simulated.

This study also identified that the number of ANC visits improves with women's educational level, and women who have attended formal education were shown to have an increased number of ANC visits compared with women who did not. Previous studies in Ethiopia also support the current finding (4,28,29). This finding is also supported by a study conducted in sub-Saharan Africa from 2008 to 2019 (28,30). Further, it is consistent with a study conducted in Bangladesh in 2020 (10) and Nigeria in 2020 (31). Similarly, another study conducted in Indonesia also identified that educated women utilize more ANC visits than women who do not have formal education (32). This might be because educated women have a higher chance of receiving information on the benefit of ANC visits because of their exposure to mass media (30). In addition, women who have formal education may know the benefit of ANC visits to health of mother and their newnborns.

ANC visits increase as the family wealth index increases from poor to rich. This study revealed that women from rich, middle-class families have a higher number of ANC compared with those from poor families. The finding aligns with previous studies conducted in Ethiopia (4,29,33). Similarly, a population-based study conducted in Guinea also reported that educated women are more likely to utilize ANC than non-educated women (34). Further, this finding is supported by a study conducted in East African countries (35) and a population-based study from Nepal (31). This might be because women from rich families were more likely to be exposed to mass media, and in addition to this, women from rich families are more likely to have access to health facilities (36).

Women from rural areas were shown to have a reduced number of ANC visits compared with urban residents. This finding is supported by different studies conducted in Ethiopia (21,29,33), Guinea(37), Pakistan (38), the Philippines, and Indonesia (39). This may be because women from rural areas have low access to health facilities and inadequate information on the importance of having an adequate number of ANC visits.

The number of ANC visits was reduced among women from the pastoralist region compared to women from the agrarian region, while it was increased among women from the metropolis region. Even though different studies conducted in Ethiopia have not reported using the current classification of regions, three studies reported that being in the dominating rural regions was less likely to have an increased number of ANC visits than the metropolis region (6,23,33). This may be because women in the pastoralist region are less likely to attend education compared with agrarian and metropolis regions.

**Conclusion**

In this study, out of the total ANC candidate women, about 26.5 % did not have ANC visits during their pregnancy, and only 3% were shown to have eight and above ANC visits. Factors like maternal age, educational level, wealth index, place of residence, and region of residence were shown to have significant associations with the number of ANC visits in Ethiopia. Therefore, improving women's education, empowering household income, and providing health education, especially for women in pastoralist regions, may significantly increase the number of ANC visits recommended by WHO; thereby, maternal and child health will be improved.

**Ethics Approval and Consent to Participate**

The Ethical clearance was provided from the Ethiopia Health and Nutrition Research Institute Review Board. Informed verbal consent was obtained from each women. The data for this study was obtained after submitting aprotocol explains the objective of the study to DHS program via online. The detail of the ethical issues has been published in the EDHS final report, which can be accessed at: http://www.dhsprogram. com.

**Availability of Supporting Data**

The data was obtained from the DHS website https://dhsprogram.com after submition of title and a detailed summary of this study.

**Author Contributions**

DA: Conceptualized, designed the study and data curation, performed the analysis, and wrote and approved the final manuscript. DA and TG: Contributed to the analysis, critically reviewed, and approved the final manuscript. VKC: Critically revised the manuscript and approved the final manuscript. All authors read and approved the final manuscript before submission.

**Funding**

The author received no specific funding for this work.

**Conflict of Interest**

The author has declared that no competing interests exist.

**Reference**

1.	Dusingizimana T, Ramilan T, Weber JL, Iversen PO. Predictors for achieving adequate antenatal care visits during pregnancy : a cross ‑ sectional study in rural Northwest Rwanda. BMC Pregnancy Childbirth [Internet]. 2023;23(69):1–9. Available from: https://doi.org/10.1186/s12884-023-05384-0

2.	Alem ZA, Yigizie Y, Liyew MA, Tesema A, Alamneh TS, Worku MG, et al. Timely initiation of antenatal care and its associated factors among pregnant women in sub-Saharan Africa : A multicountry analysis of Demographic and Health Surveys. PLoS One [Internet]. 2022;17(1):1–17. Available from: http://dx.doi.org/10.1371/journal.pone.0262411

3.	Ambaye E, Regasa ZW, Hailiye G. Early initiation of antenatal care and its associated factors among pregnant women attending antenatal care at public health centres in Bahir Dar Zuria zone , Northwest Ethiopia , 2021 : a cross- ­ sectional study. BMJ Open. 2023;13:1–7.

4.	Yemane GD. The factors associated with antenatal care utilization in Ethiopia. Ann Med Surg [Internet]. 2022;79(June):104092. Available from: https://doi.org/10.1016/j.amsu.2022.104092

5.	Raru TB, Ayana GM, Bahiru N, Deressa A, Alemu A, Birhanu A, et al. Quality of antenatal care and associated factors among pregnant women in East Africa using Demographic and Health Surveys : A multilevel analysis. Women’s Heal. 2022;18(1–9).

6.	Shibre G, Zegeye B, Idriss-wheeler D, Ahinkorah BO. Socioeconomic and geographic variations in antenatal care coverage in Angola : further analysis of the 2015 demographic and health survey. BMC Public Health. 2020;20(12243):1–10.

7.	Raru TB, Ayana GM, Zakaria HF, Merga BT. Association of Higher Educational Attainment on Antenatal Care Utilization Among Pregnant Women in East Africa Using Demographic and Health Surveys ( DHS ) from 2010 to 2018 : A Multilevel Analysis. Int J ofWomen’s Heal. 2022;14(January):67–77.

8.	Muluneh AG, Animut Y, Ayele TA. Spatial clustering and determinants of home birth after at least one antenatal care visit in Ethiopia : Ethiopian demographic and health survey 2016 perspective. BMC Pregnancy Childbirth. 2020;20(97):1–13.

9.	Rurangirwa AA, Mogren I, Nyirazinyoye L, Ntaganira J, Krantz G. Determinants of poor utilization of antenatal care services among recently delivered women in Rwanda ; a population based study. BMC Pregnancy Childbirth. 2017;17(142):1–10.

10.	Islam A, Kabir R, Talukder A. Triggering factors associated with the utilization of antenatal care visits in Bangladesh : An application of negative binomial regression model. Clin Epidemiol Glob Heal [Internet]. 2020;8(4):1297–301. Available from: https://doi.org/10.1016/j.cegh.2020.04.030

11.	Sisay G, Mulat T. Antenatal Care Dropout and Associated Factors in Ethiopia : A Systematic Review and Meta-Analysis Authors Name : Gizaw Sisay 1 Tsion Mulat 1 Authors affiliation : Correspondence : Conclusion : 2023;1–22.

12.	Andegiorgish AK, Elhoumed M, Qi Q. Determinants of antenatal care use in Saharan African countries : a nine sub- ­ statistical analysis of cross- ­ sectional data from Demographic and Health Surveys. BMJ Open. 2022;12:1–13.

13.	Tessema ZT, Tesema GA. Individual- ­ level and community- ­ level factors associated with eight or more Saharan antenatal care contacts in sub- ­ Africa : evidence from 36 sub- ­ Saharan African countries. BMJ Open. 2022;12:1–8.

14.	Shiferaw K, Mengistie B, Gobena T, Dheresa M, Seme A. Extent of Received Antenatal Care Components in Ethiopia : A Community-Based Panel Study. Int J Womens Health. 2021;13:803–13.

15.	Tegegne TK, Chojenta C, Getachew T, Smith R, Loxton D. Antenatal care use in Ethiopia : a spatial and multilevel analysis. BMC Pregnancy Childbirth. 2019;19(299):1–16.

16.	Muluneh AA, Kassa ZY, Mamo ZB. UTILIZATION OF ANTENATAL CARE AND ASSOCIATED FACTORS IN. Ethiop J Reprod Heal. 2021;13(1).

17.	Tura G. ORIGI AL ARTICLE A TE ATAL CARE SERVICE UTILIZATIO A D ASSOCIATED FACTORS I METEKEL ZO E , ORTHWEST ETHIOPIA. Ethiop J Heal Sci. 2009;19(2).

18.	Debelo BT, Danusa KT. Level of Late Initiation of Antenatal Care Visit and Associated Factors Amongst Antenatal Care Attendant Mothers in Gedo General Hospital , West Shoa Zone , Oromia Region ,. Front Public Heal. 2022;10(June):1–8.

19.	Yezengaw TY. Late Initiation of Antenatal Care and Associated Factors among Antenatal Care Attendees in Governmental Health Centers of Harar. J womens Heal care. 2022;11:1–10.

20.	Weldearegawi GG, Teklehaimanot BF, Gebru HT. Determinants of late antenatal care follow up among pregnant women in Easter zone Tigray , Northern Ethiopia , 2018 : unmatched case – control study. BMC Res Notes [Internet]. 2019;1–9. Available from: https://doi.org/10.1186/s13104-019-4789-8

21.	Tesfaye M, Dessie Y, Demena M. Late antenatal care initiation and its contributors among pregnant women at selected public health institutions in Southwest Ethiopia Article Abstract. 2021;

22.	Central statistical Agency. Population Size by Sex, Area and Density by Region, Zone and Wereda. 2022.

23.	Ethiopian Public Health Institute (EPHI). Ethiopia Mini Demographic and Health Survey 2019: Final Report [Internet]. 2021. Available from: https://dhsprogram.com/pubs/pdf/FR363/FR363.pdf

24.	USAID. Guide to DHS Statistics. Vol. 7. 2018.

25.	Theall KP, Scribner R, Broyles S, Yu Q, Chotalia J, Simonsen N, et al. Impact of small group size on neighbourhood influences in multilevel models. J Epidemiol Community Heal. 2011;4(65):688–95.

26.	Hassen SS, Teshale BM, Adulo LA. Identifying Factors Associated with Barriers in the Number of Antenatal Care Service Visits among Pregnant Women in Rural Parts of Ethiopia. Sci World J. 2021;2021.

27.	Kuuire VZ, Kangmennaang J, Atuoye KN, Antabe R, Boamah SA, Vercillo S, et al. Timing and utilisation of antenatal care service in Nigeria and Malawi. Glob Public Health [Internet]. 2017;1692. Available from: https://doi.org/10.1080/17441692.2017.1316413

28.	Aderajew M, Id Z. Number of antenatal care visits and associated factors among reproductive age women in Sub-Saharan Africa using recent demographic and health survey data from 2008 – 2019 : A multilevel negative binomial regression model. PLOS Glob PUBLIC Heal [Internet]. 2022;83:1–14. Available from: http://dx.doi.org/10.1371/journal.pgph.0001180

29.	Tessema ZT, Akalu TY. Spatial Pattern and Associated Factors of ANC Visits in Ethiopia: Spatial and Multilevel Modeling of Ethiopian Demographic Health Survey Data. Adv Prev Med. 2020;2020:1–13.

30.	Tessema ZT, Teshale AB, Tesema GA. Determinants of completing recommended antenatal care utilization in sub-Saharan from 2006 to 2018 : evidence from 36 countries using Demographic and Health Surveys. BMC Pregnancy Childbirth. 2021;21(192):1–12.

31.	El-Khatib Z, Odusina EK, Ghose B, Yaya S. Patterns and predictors of insufficient antenatal care utilization in Nigeria over a decade: A pooled data analysis using demographic and health surveys. Int J Environ Res Public Health. 2020;17(21):1–14.

32.	Laksono AD, Rukmini R, Wulandari RD. Regional disparities in antenatal care utilization in Indonesia. PLoS One [Internet]. 2020;15(2):1–13. Available from: http://dx.doi.org/10.1371/journal.pone.0224006

33.	Arefaynie M, Kefale B, Yalew M, Adane B, Dewau R, Damtie Y. Number of antenatal care utilization and associated factors among pregnant women in Ethiopia : zero ‑ inflated Poisson regression of 2019 intermediate Ethiopian Demography Health Survey. Reprod Health [Internet]. 2022;19(36):1–10. Available from: https://doi.org/10.1186/s12978-022-01347-4

34.	Shibre G, Zegeye B, Idriss-wheeler D. Factors affecting the utilization of antenatal care services among women in Guinea : a population-based study. Heal Serv Res Factors. 2020;(4):1–7.

35.	Tessema ZT. Utilization and Determinants of Antenatal Care Visits in East African Countries : A Multicountry Analysis of Demographic and. Adv Med Stat. 2021;2021.

36.	Anaba EA, Alor SK, Badzi CD. Utilization of antenatal care among adolescent and young mothers in Ghana; analysis of the 2017/2018 multiple indicator cluster survey. BMC Pregnancy Childbirth [Internet]. 2022;22(1):1–8. Available from: https://doi.org/10.1186/s12884-022-04872-z

37.	Seidu A. Public Health in Practice A multinomial regression analysis of factors associated with antenatal care attendance among women in Papua New Guinea. Public Heal Pract [Internet]. 2021;2(February):100161. Available from: https://doi.org/10.1016/j.puhip.2021.100161

38.	Ali SA, Ali SA, Feroz A, Saleem S, Fatmai Z, Kadir MM. Factors affecting the utilization of antenatal care among married women of reproductive age in the rural Thatta , Pakistan : findings from a community-based case-control study. BMC Pregnancy Childbirth. 2020;20(355):1–12.

39.	Wulandari RD, Laksono AD, Rohmah N. Urban-rural disparities of antenatal care in South East Asia : a case study in the Philippines and Indonesia. 2021;1–9.
